# Supplementary material for: Promiscuity in Polyphenol–Protein Interactions—Monitoring Protein Conformational Change upon Polyphenol–Protein Binding by Nano-Differential Fluorimetry (Nano-DSF)
Source: Molecules. 2025 Feb 19;30(4):965. doi: 10.3390/molecules30040965 (PMC11858516; doi:10.3390/molecules30040965)
Supplement: Supplementary file 1 [file molecules-30-00965-s001.zip › molecules-3398451-supplementary.pdf]

Supplementary information

Investigation of Polyphenol Protein Interactions using nano Differential Fluorimetry

Maria Bikaki, Lijing Jao, Paula Cotrell, Anastasiia Shevchuk and Nikolai Kuhnert*

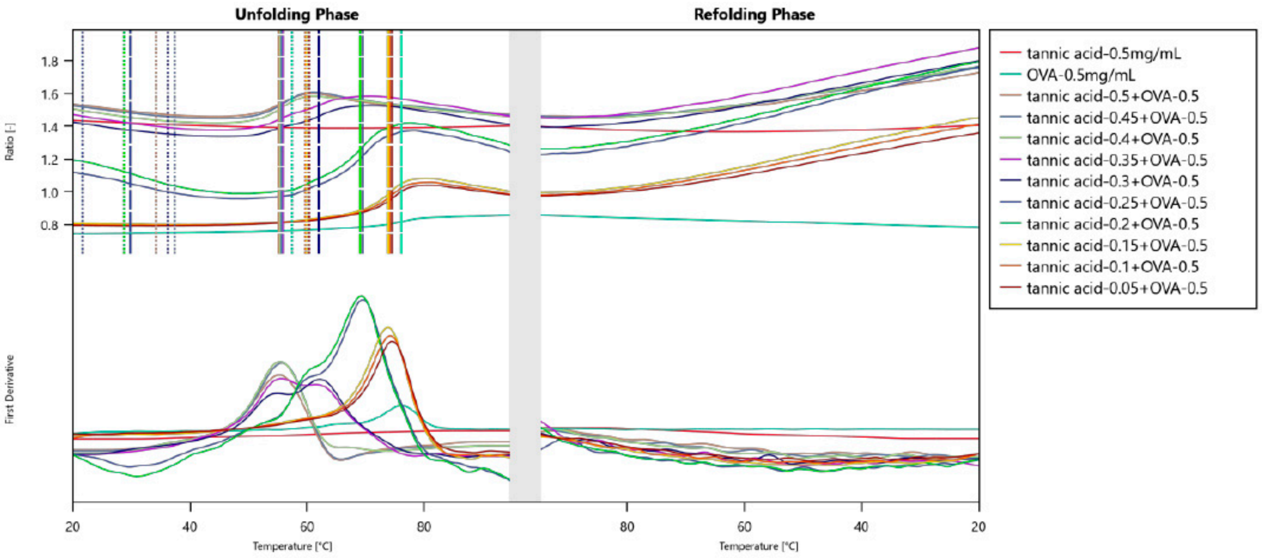

Figure S1: Nano DSF measurement of Unfolding and refolding phases of the interaction between OVA and tannic acid. [OVA] 2.92 μM; [tannic acid] 0-146.96 μM with increasing temperature (left panel) and decreasing temperature (right panel). Upper panels show F320/350 nm ratio and lower panels its first derivative.

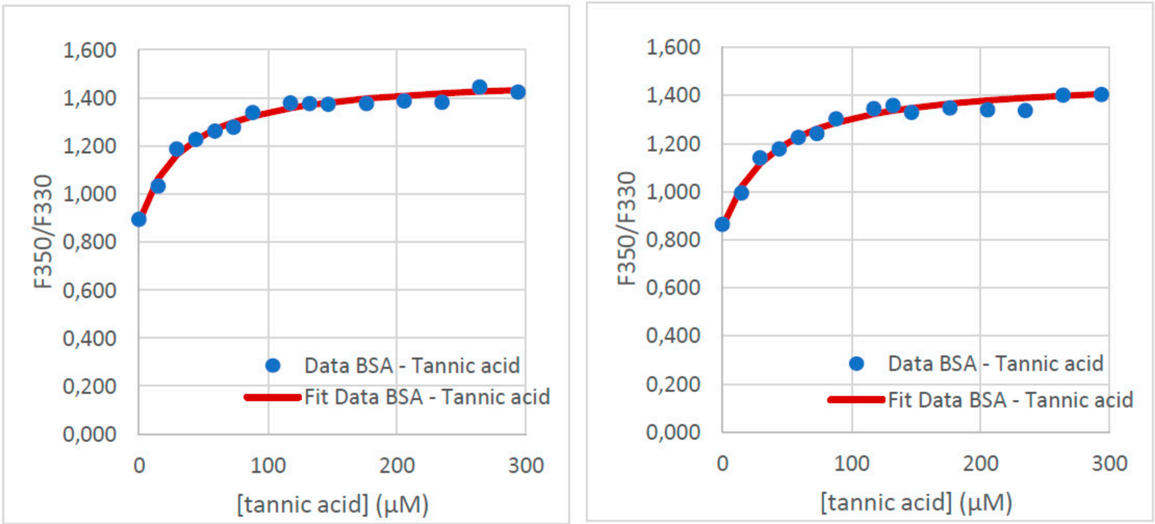

Figure S2: Determination of K_a for the binding of tannic acid to BSA using the ratio of fluorescence intensity at 350 nm to 330 nm at 20 °C (left) and 37 °C (right).
[BSA] 3.76 μM; [tannic acid] 0-293.91 μM; model = one site binding.

Left: SSD = 0.003; K_a = 29878.34M^{-1}. Right: SSD = 0.004; K_a = 26269.37 M^{-1}.

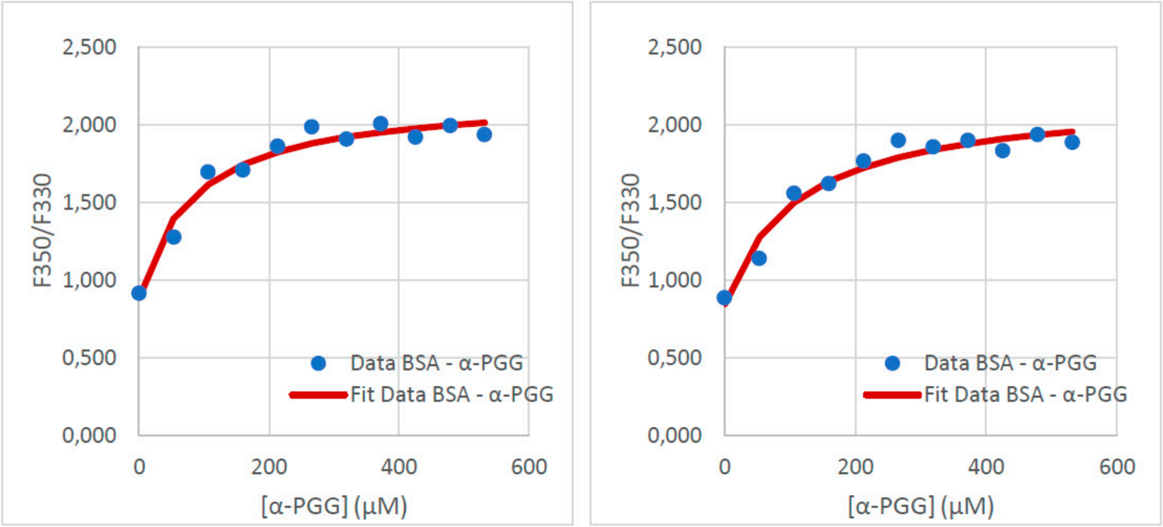

Figure S3: Determination of K_a for the binding of α-PGG to BSA using the ratio of fluorescence intensity at 350 nm to 330 nm at 20 °C (left) and 37 °C (right).
[BSA] 0.75 μM; [α-PGG] 0-531.85 μM; model = one site binding.
Left: SSD = 0.047; K_a = 1890.53 M^{-1}. Right: SSD = 0.049; K_a = 8942.56 M^{-1}.

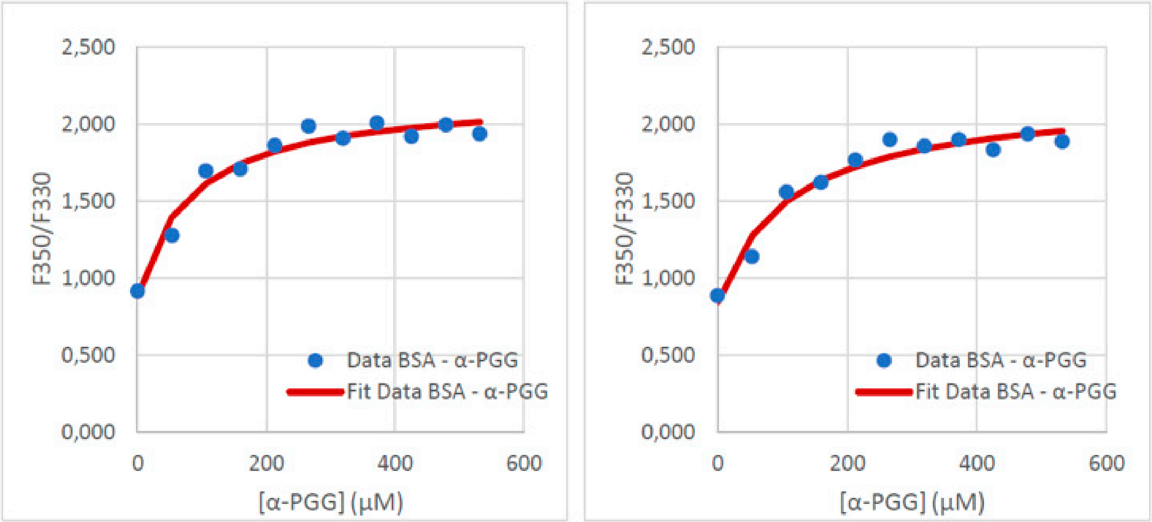

Figure S4: Determination of K_a for the binding of β-PGG to BSA using the ratio of fluorescence intensity at 350 nm to 330 nm at 20 °C (left) and 37 °C (right).
[BSA] 0.75 μM; [β-PGG] 0-531.85 μM; model=one site binding.
Left: SSD = 0.027; K_a = 13392.23 M^{-1}. Right: SSD = 0.040; K_a = 11196.00 M^{-1}.

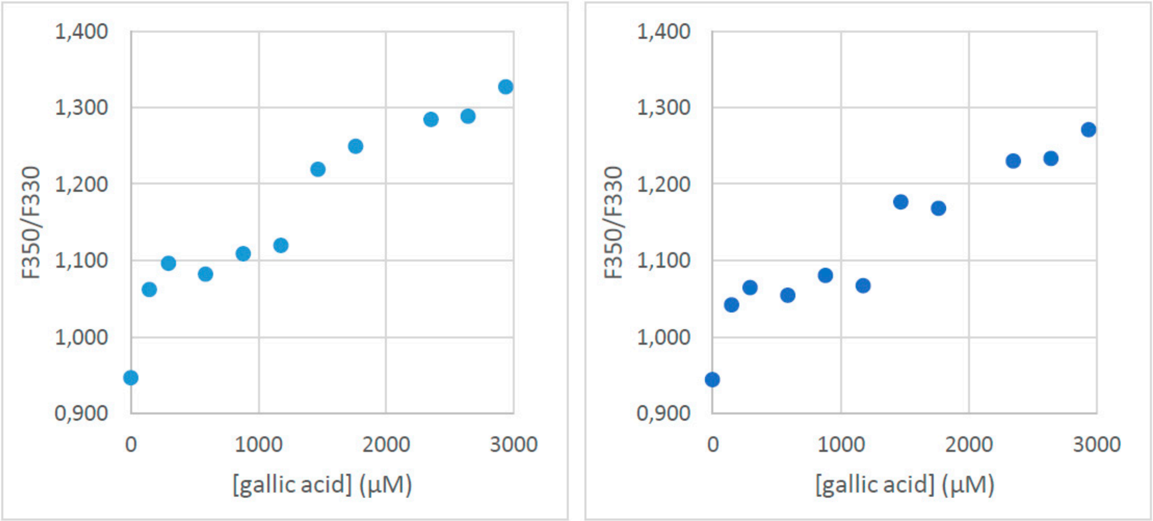

Figure S5: Binding curve indicating the interaction between gallic acid and BSA using the ratio of fluorescence intensity at 350 nm to 330 nm at 20 °C (left) and 37 °C (right).
[BSA] 0.75 µM; [gallic acid] 0-2939.10 µM.

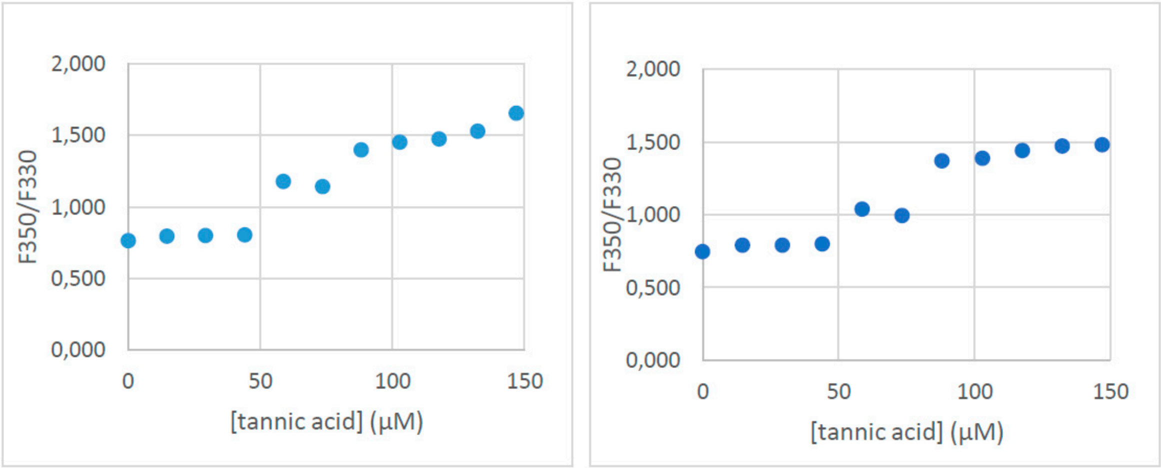

Figure S6: Binding curve showing the interaction between tannic acid and OVA using the ratio of fluorescence intensity at 350 nm to 330 nm at 20 °C (left) and 37 °C (right).
[OVA] 5.83 µM; [tannic acid] 0-146.96 µM.

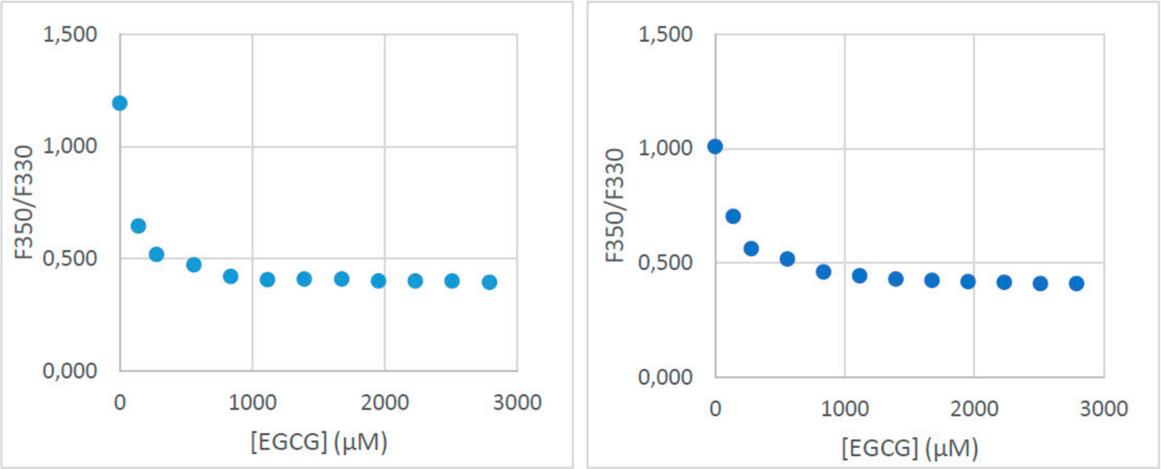

Figure S7: Binding curve of the interaction between EGCG and pepsin using the ratio of fluorescence intensity at 350 nm to 330 nm at 20 °C (left) and 37 °C (right).
[pepsin] 2.42 µM; [EGCG] 0-2790.40 µM.

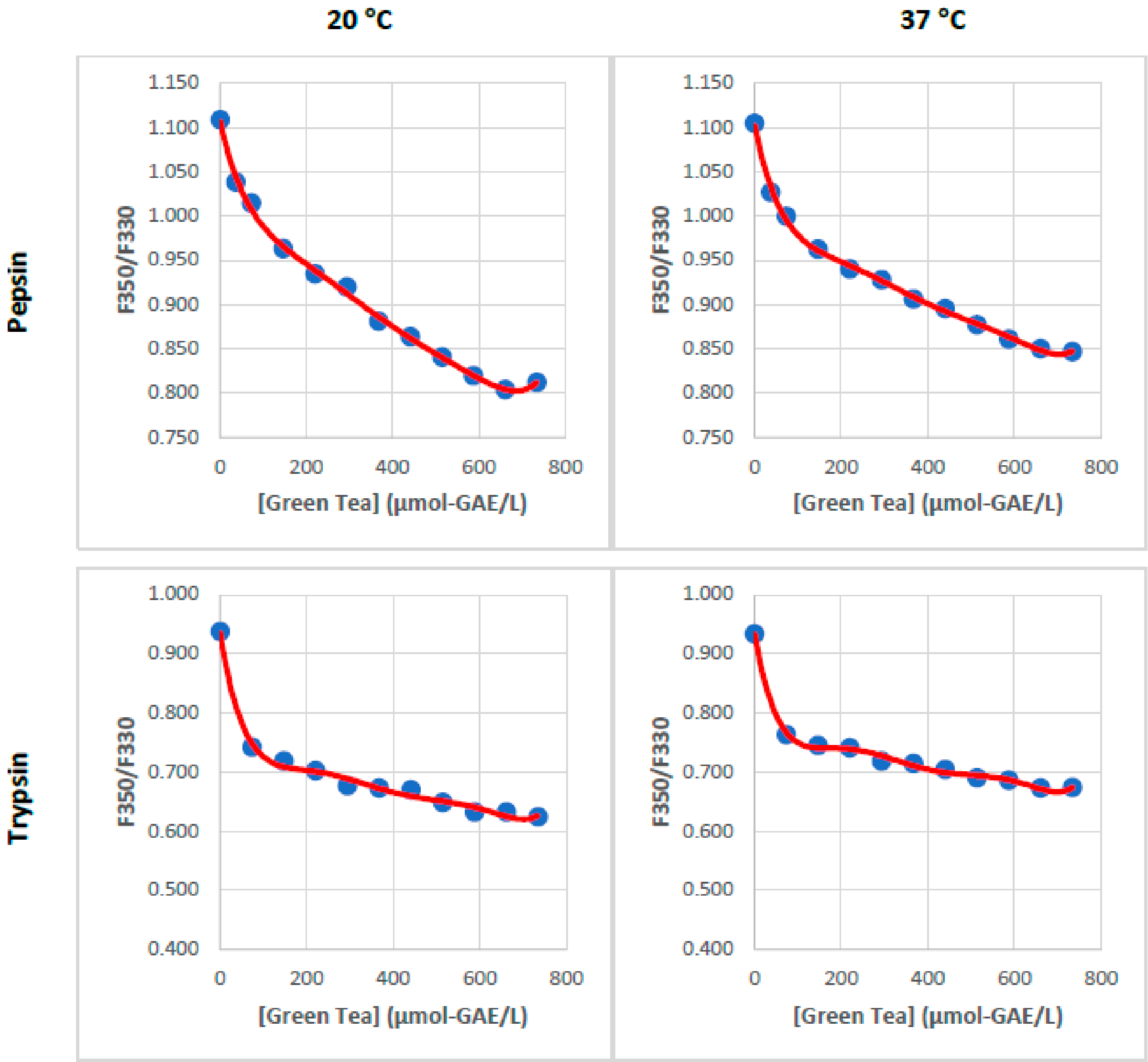

Figure S8: Binding curve of green tea to pepsin, trypsin and ACE at the temperature points of 20 °C (left) and 37 °C (right). While the green tea concentration used was kept at a constant range of 0-733.41µM, the pepsin, trypsin and ACE proteins were used at a concentration of 6.05 µM, 10.73 µM and 0.36 µM (respectively).

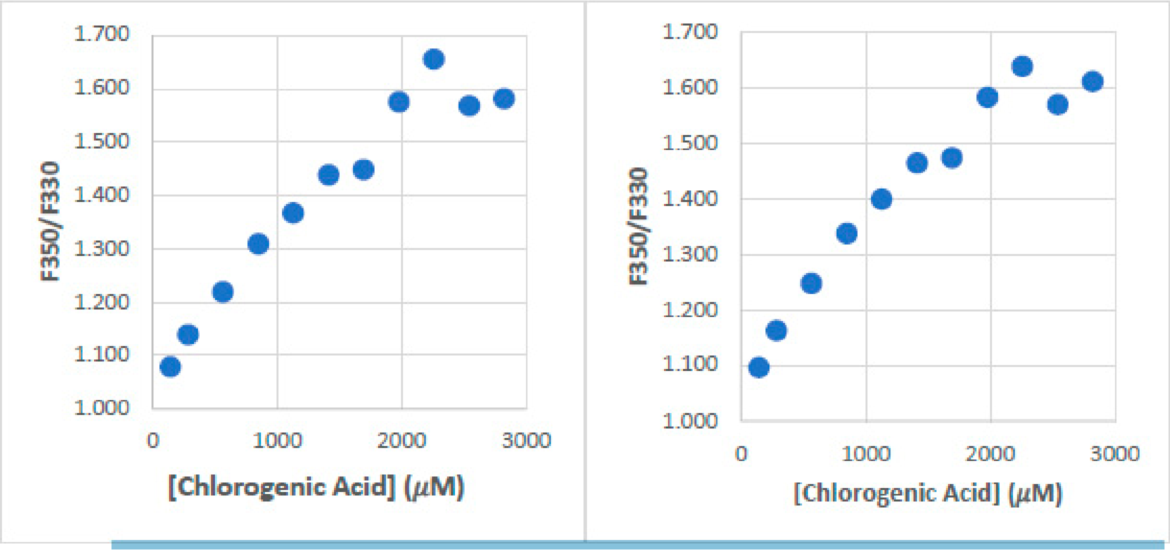

Figure S9: The binding curve of the interaction between CGA and pepsin denoted by the fluorescence ratio (F350/F330) at 20 °C (left) and 37 °C (right). The concentration of pepsin and chlorogenic acid used were 6.06 µM and 0-2822.39 µM (respectively).

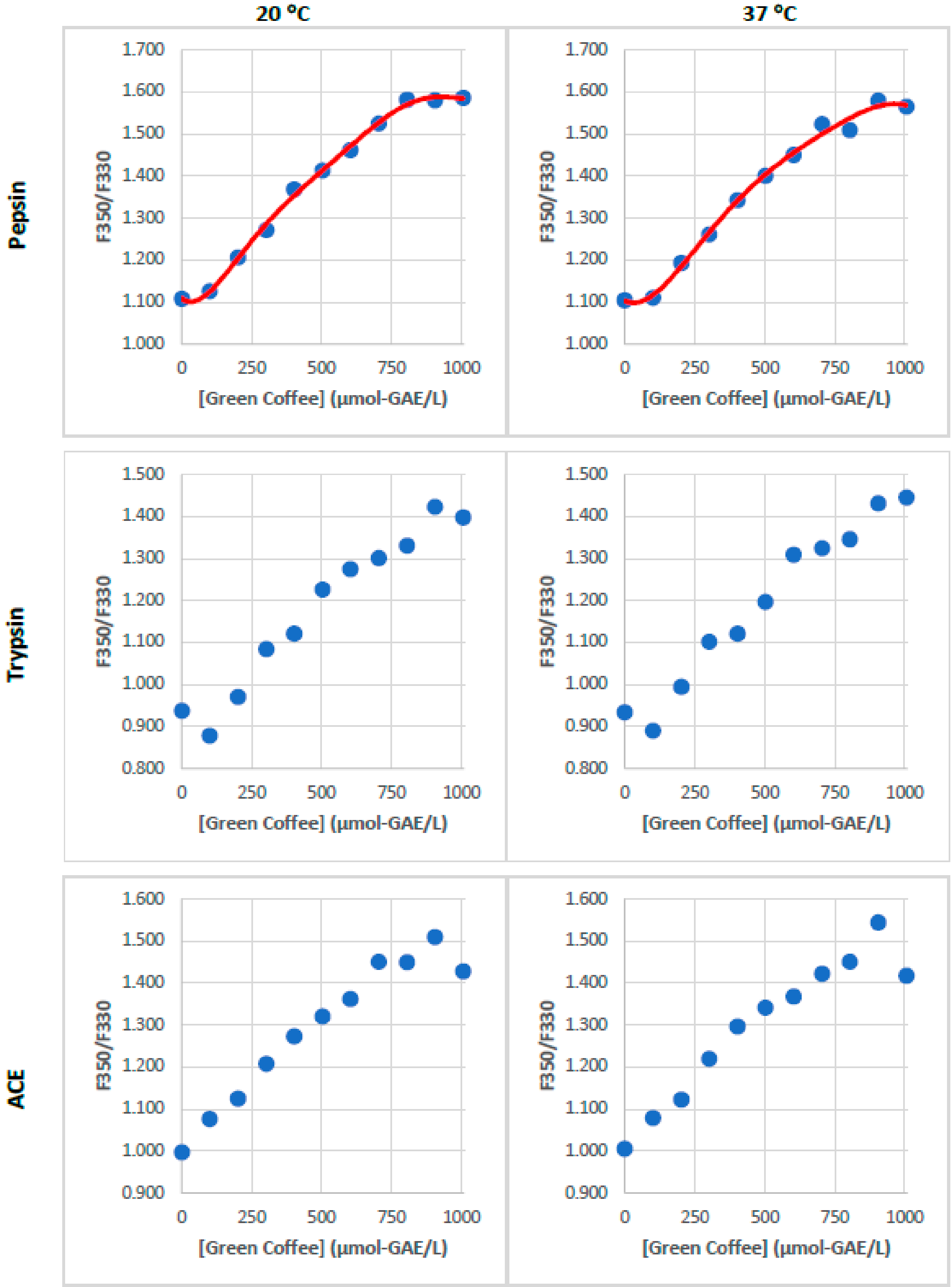

Figure S10: Binding curve of green coffee to pepsin (top), trypsin (middle) and ACE (bottom) at the temperaturepoints of 20 °C (left) and 37 °C (right). While the green coffee concentration used was kept at a constant rangeof 0-1006.04 µmol-GAE/L, the pepsin, trypsin and ACE proteins were used at a concentration of 6.05 µM, 10.73µM and 0.36 µM (respectively).

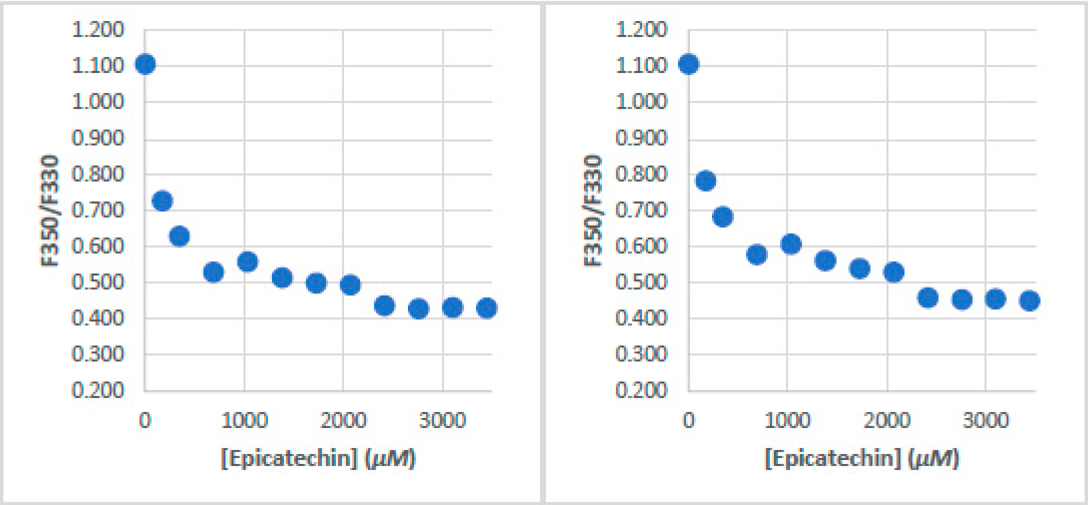

Figure S11: The binding curve of the interaction between epicatechin and pepsin denoted by the fluorescence ratio (F350/F330) at 20 °C (left) and 37 °C (right). The concentration of pepsin and epicatechin used were 6.06 µM and 0-3445.07 µM (respectively).

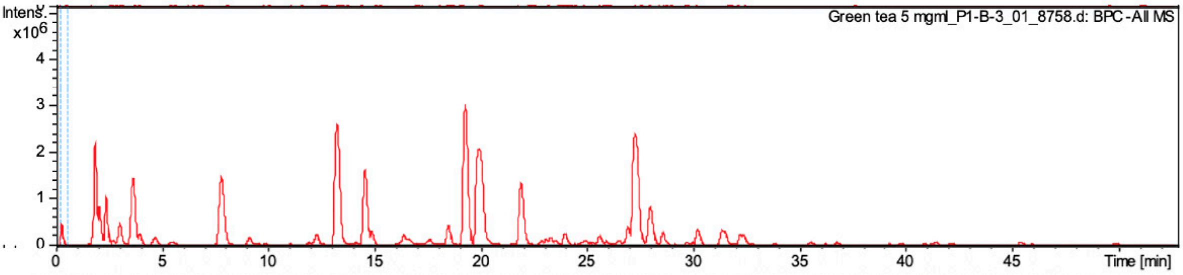

Figure S12: Negative ion mode chromatogram of green tea extract employed in nano DSF titrations. For chromatographic method please see Shevchuk et al 2019.

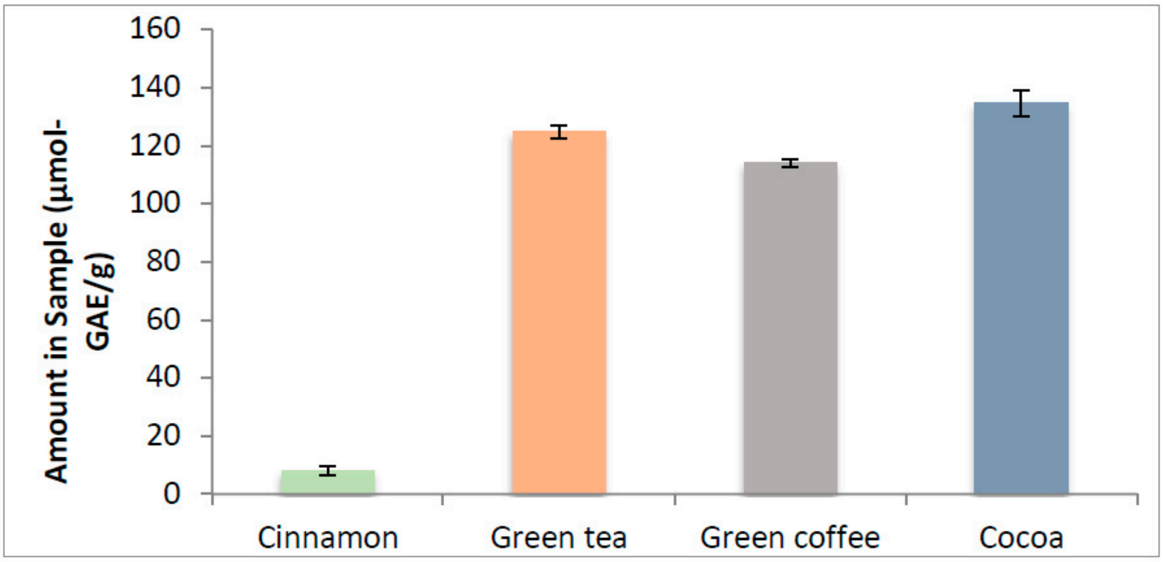

Figure S13: Results of FRAP assay to determine antioxidant capacity of dietary extracts employed in nano DSF titrations.

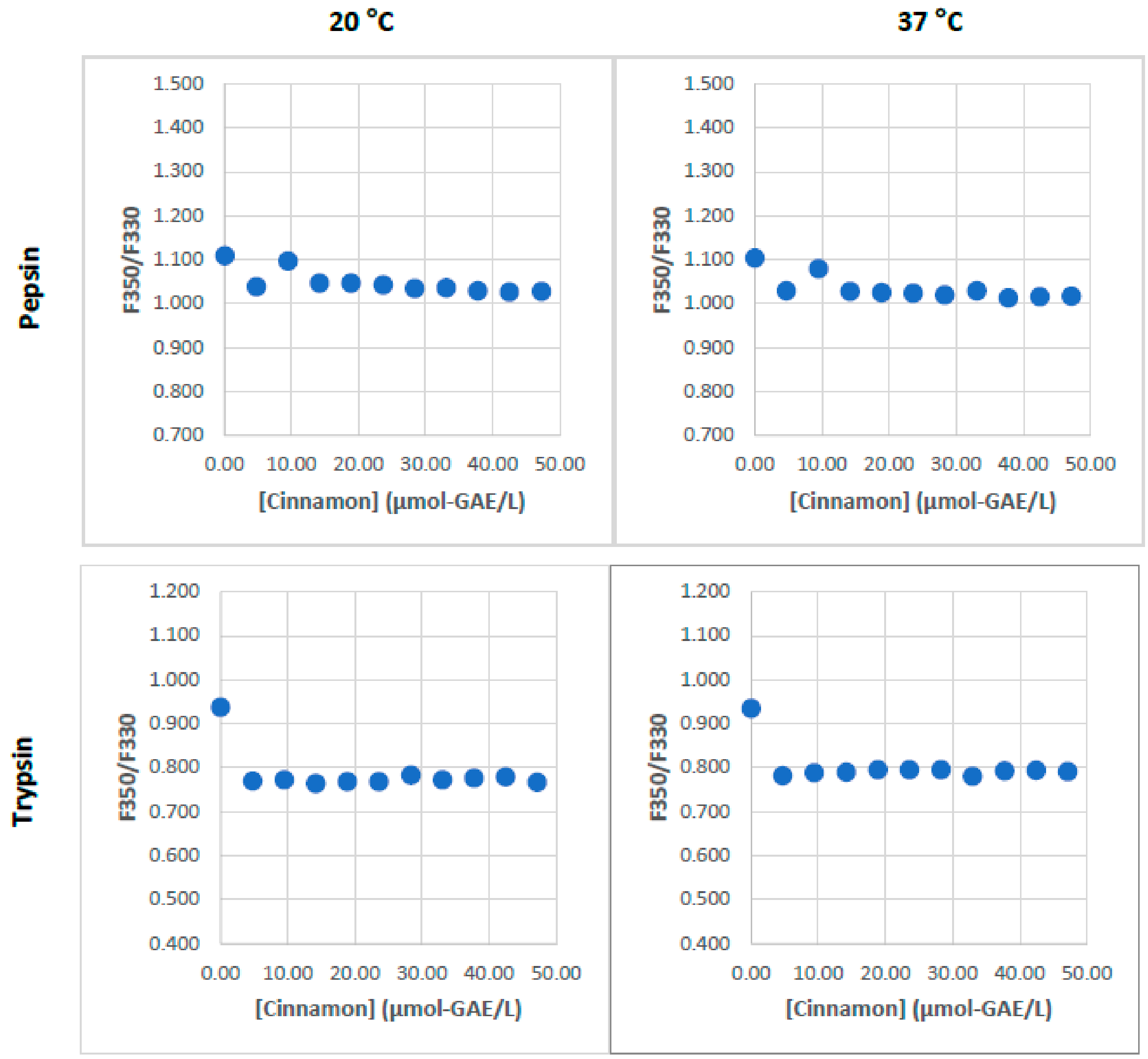

Figure S14 :Binding curve of cinnamon to pepsin (top), trypsin (middle) and ACE (bottom) at the temperature points of 20 °C (left) and 37 °C (right). While the cinnamon concentration used was kept at a constant range of 0-47.18 µM-GAE/L, the pepsin, trypsin and ACE proteins were used at a concentration of 6.05 µM, 10.73 µM and 0.36 µM (respectively).
